# A delay in vesicle endocytosis by a C-terminal fragment of N-cadherin enhances Aβ synaptotoxicity

**DOI:** 10.1038/s41420-023-01739-w

**Published:** 2023-12-08

**Authors:** Zenghui Teng, Georgia-Ioanna Kartalou, Sushma Dagar, Patrick C. Fraering, Volkmar Lessmann, Kurt Gottmann

**Affiliations:** 1 Institute of Neuro- and Sensory Physiology, Medical Faculty, Heinrich-Heine-University Düsseldorf, Düsseldorf, Germany; 2 Institute of Physiology, Medical Faculty, Otto-von-Guericke-University Magdeburg, Magdeburg, Germany; 3 Foundation Eclosion, CH1228 Plan-les-Ouates & Campus Biotech Innovation Park, CH1202 Geneva, Switzerland; 4 Center for Behavioral Brain Sciences, Otto-von-Guericke-University Magdeburg, Magdeburg, Germany

**Keywords:** Alzheimer's disease, Cellular neuroscience

## Abstract

Synaptotoxic Aβ oligomers are thought to play a major role in the early pathology of Alzheimer´s disease (AD). However, the molecular mechanisms involved in Aβ-induced synaptic dysfunction and synapse damage remain largely unclear. Previously, Aβ synaptotoxicity has been reported to be enhanced by increased levels of a C-terminal fragment of the synaptic adhesion molecule N-cadherin that is generated by proteolytic shedding of the extracellular domains [1]. To address the molecular mechanisms involved in this process, we have now studied the functional synaptic changes induced by C-terminal fragments (CTF1) of synaptic adhesion proteins. We used synaptophysin-pHluorin (SypHy) fluorescence imaging to monitor synaptic vesicle exo- and endocytosis in cultures of mouse cortical neurons. We increased the levels of C-terminal fragments of synaptic adhesion proteins by pharmacologically inhibiting γ-secretase, which further degrades CTF1 fragments. We found that this intervention caused a delay in synaptic vesicle endocytosis. A similar effect was induced by overexpression of N-cadherin CTF1, but not by overexpression of Neurexin3β CTF1. Based on these observations, we further studied whether directly modulating synaptic vesicle endocytosis enhances Aβ synaptotoxicity. We pharmacologically induced a delayed synaptic vesicle endocytosis by a low concentration of the endocytosis inhibitor dynasore. This treatment enhanced synaptoxicity of Aβ oligomers as indicated by a reduced frequency of miniature postsynaptic currents. In conclusion, we propose that delayed endocytosis results in prolonged exposure of synaptic vesicle membranes to the extracellular space, thus enabling enhanced vesicle membrane binding of Aβ oligomers. This might in turn promote the endocytic uptake of toxic Aβ oligomers and might thus play an important role in intracellular Aβ-mediated synaptotoxicity in AD.

## Introduction

Alzheimer´s disease (AD) is a complex neurodegenerative disorder that progresses from mild memory impairment to massive cognitive deficits and strong brain atrophy [2–4]. AD involves several brain cell types including microglia, and a variety of molecular mechanisms with amyloid-β (Aβ) and tau pathology being the best characterized [5, 6]. At early stages of AD, synaptotoxic Aβ oligomers are thought to be of major importance by affecting synaptic plasticity and synapse stability [7–10].

Extracellular addition of synaptotoxic Aβ oligomers is well known to inhibit hippocampal long-term potentiation (LTP) [11, 12], and thereby might lead to episodic memory impairment in vivo. Mechanistically, extracellular Aβ oligomers have been suggested to induce LTD-like mechanisms including activation of extrasynaptic NMDA receptors and endocytosis of postsynaptic AMPA receptors [13–17] thus counteracting synaptic AMPA receptor insertion needed for LTP. A number of candidate surface membrane proteins have been suggested to function as native Aβ receptors [16, 18].

In addition to extracellular Aβ oligomers, intracellular Aβ has also been proposed to play an important role in damaging subcellular organization [19–22]. Importantly, intracellular Aβ has been suggested to induce impaired mitophagy leading to the accumulation of defective mitochondria at presynaptic sites [23, 24]. This might in turn lead to presynaptic degeneration and synapse loss [25]. Uptake of extracellular Aβ oligomers by endocytosis might be crucial for accumulating toxic amounts of Aβ intracellularly [22, 26].

Synaptotoxic effects of Aβ oligomers are thought to strongly depend on the cellular and molecular context thus resulting in subsets of highly vulnerable cells and synapses. In our previous work [1], we identified a proteolytic C-terminal fragment of the synaptic adhesion protein N-cadherin (N-cadherin CTF1) [27] as a molecular factor strongly enhancing Aβ synaptotoxicity. However, the molecular mechanism underlying this phenomenon remained elusive. In the present work, we studied the effects of increased levels of different adhesion protein CTFs, and found that selectively N-cadherin CTF1 was inducing a delay in synaptic vesicle endocytosis. Slower vesicle endocytosis might lead to increased vesicle membrane binding and uptake of Aβ, and might thus enhance the accumulation of intracellular Aβ. This mechanism was corroborated by an enhancement of synaptotoxic effects of Aβ oligomers upon delaying vesicle endocytosis by a low concentration of the endocytosis inhibitor dynasore.

## Results

### Pharmacological inhibition of γ-secretase slows synaptic vesicle endocytosis

**Fig. 1:**
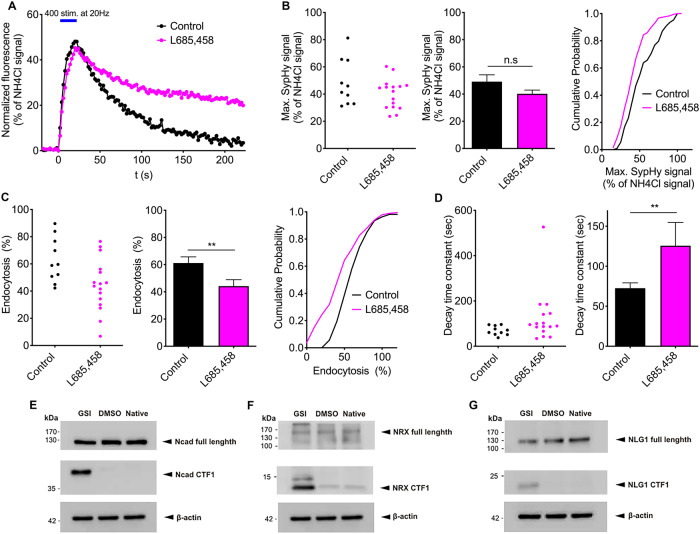
Pharmacological inhibition of γ-secretase slows synaptic vesicle endocytosis. **A** Synaptophysin-pHluorin (SypHy) imaging of autapses in microisland cultures of cortical neurons reveals synaptic vesicle exo- and endocytosis elicited by electrical stimulation (400 stimuli at 20 Hz; indicated by blue bar). Time courses of SypHy fluorescence in control (black trace) and L-685,485 (γ-secretase inhibitor, 5 µM for 2 days; magenta trace) treated neurons. Individual SypHy puncta on a transfected neuron were averaged per cell and normalized to the NH_4_Cl signal. **B** The maximal SypHy fluorescence signal did not significantly differ between control and L-685,485 treated neurons. Left and middle panel: SypHy puncta of each cell (control: *n* = 10; L-685,485 treated: *n* = 16) were averaged. Individual values for each cell (left) and mean ± SEM (middle) are shown. Right panel: Cumulative distributions of individual SypHy puncta of all cells recorded (control: *n* = 108 puncta; L-685,485: *n* = 185 puncta). **C** Percent loss of SypHy signal (% endocytosis) 90 s after the end of stimulation was significantly reduced in L-685,485 treated neurons. Left and middle panel: SypHy puncta of each cell were averaged. Right panel: Cumulative distributions of individual SypHy puncta of all cells recorded. **D** The decay time constant of SypHy fluorescence decay was significantly increased in L-685,485 treated neurons. Monoexponential fit of the average time course of SypHy signals from all puncta of a given cell (**A**) was used. Individual values for each cell (left) and mean ± SEM (right) are shown. Student’s *t* test; **P < 0.01; n.s. non significant. **E**–**G** Western blot analysis of the major sheddase-dependent C-terminal fragments (CTF1) of the indicated synaptic adhesion proteins upon pharmacological inhibition of γ-secretase in cultured hippocampal neurons. Note the strong increases in CTF1 fragments of N-cadherin (**E**, Ncad-CTF1), neurexins (**F**, NRX-CTF1; anti-Neurexin 1/2/3 antibody), and neuroligin1 (**G**, NLG1-CTF1) following treatment with the γ-secretase inhibitor L-685,485 (GSI). DMSO: vehicle control. Native: no treatment. β-actin was used as loading control.

In a first set of experiments, we studied the effects of inhibition of γ-secretase activity by the selective inhibitor L-685,458 (5 µM) on the kinetics of synaptic vesicle endocytosis in mouse cortical neurons in microisland cultures at 12–14 DIV (Supplementary Fig. 1A). After 2 days treatment with L-685,458 electrical stimulation-induced synaptic vesicle exo- and endocytosis was quantitatively monitored by using Synaptophysin-pHluorin (SypHy) fluorescence imaging [28, 29] (Fig. 1A). We focused on the quantitative analysis of autaptic sites that were identified by localization of the fluorescent SypHy puncta on the dendrites of the same neuron (co-transfectd with SypHy and DsRed2 as transfection marker; Supplementary Fig. 1B). The maximal SypHy fluorescence increase at the end of the electrical stimulation (400 stimuli at 20 Hz) did not significantly differ between control neurons and L-685,458 treated neurons (Fig. 1B). We next analyzed the synaptic vesicle endocytosis-related SypHy fluorescence decay following the end of stimulation. Intriguingly, 90 s after the end of stimulation the relative SypHy signal loss - reflecting endocytosis - was significantly reduced in L-685,485 treated neurons (Fig. 1C). This indicates a slowing of synaptic vesicle endocytosis upon specific inhibition of γ-secretase activity. To corroborate this finding, we analyzed the SypHy fluorescence decay kinetics by monoexponential fitting. As expected, the mean decay time constant was significantly increased in L-685,485-treated neurons (Fig. 1D) thus confirming that the slowing of synaptic vesicle endocytosis was caused by inhibition of γ-secretase activity.

Several types of transsynaptic adhesion proteins (e.g., N-cadherin, neurexins, neuroligins) have previously been reported to be sequentially processed by α-secretase and γ-secretase activities. Based on these observations, blocking γ-secretase actvity by L-685,485 is expected to lead to an accumulation of C-terminal transmembrane fragments (CTF1) that are generated by cleavage of full-length proteins by α-secretase. To confirm this experimentally, we performed Western blot analyses to detect the CTF1 of N-cadherin, neurexins, and neuroligin1 (Fig. 1E–G) in cultured hippocampal neurons. As expected, the specific CTF1 of all three adhesion proteins tested (N-cadherin, neurexins, neuroligin1) were clearly increased upon inhibition of γ-secretase activity. Because these synaptic adhesion proteins play important roles in regulating synaptic functions, the increased CTF1 levels of all three adhesion proteins might potentially underly the delay of synaptic vesicle endocytosis that we observed following γ-secretase inhibition.

### Overexpression of N-cadherin-CTF1, but not of Neurexin3β-CTF1, slows synaptic vesicle endocytosis comparable to γ-secretase inhibition

We previously described that overexpression of N-cadherin-CTF1 in cultured cortical neurons leads to a delay in synaptic vesicle endocytosis (Fig. 4 in Dagar et al., 2021). In this new study, we aimed to determine whether this effect can account for the in the present paper observed slowing of synaptic vesicle endocytosis by γ-secretase inhibition. We further wanted to investigate, whether increases in other synaptic adhesion protein CTFs might also be involved in changes in synaptic vesicle endocytosis.

**Fig. 2:**
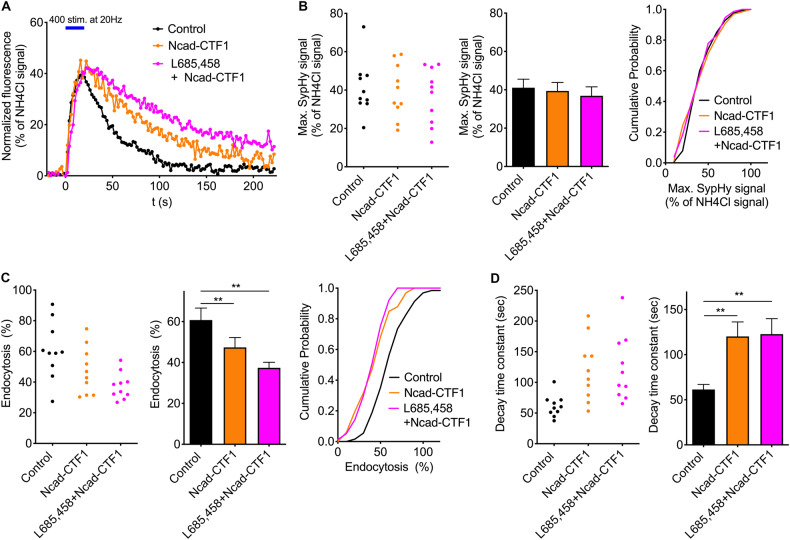
Overexpression of N-cadherin-CTF1 slows synaptic vesicle endocytosis. **A** Time courses of SypHy fluorescence changes (elicited by 400 stimuli at 20 Hz; indicated by blue bar) in control neurons (black trace), in N-cadherin-CTF1 expressing neurons (orange trace), and in N-cadherin-CTF1 expressing neurons with addition of L-685,485 (5 µM for 2 days; magenta trace). Individual SypHy puncta on a given neuron were averaged per cell and normalized to the NH_4_Cl signal. **B** The maximal SypHy fluorescence signal did not significantly differ between control and N-cadherin-CTF1 expressing neurons. Left and middle panel: SypHy puncta of each cell (control: *n* = 10; N-cadherin-CTF1 expressing: *n* = 10; N-cadherin-CTF1 expressing with addition of L-685,485: *n* = 10) were averaged. Individual values for each cell (left) and mean ± SEM (middle) are shown. Right panel: Cumulative distributions of individual SypHy puncta of all cells recorded (control: *n* = 128 puncta; N-cadherin-CTF1 expressing: *n* = 66 puncta; N-cadherin-CTF1 expressing with addition of L-685,485: *n* = 77 puncta). **C** The percent loss of the SypHy signal (% endocytosis) 90 s after end of stimulation was significantly reduced in N-cadherin-CTF1 expressing neurons. Left and middle panel: SypHy puncta of each cell were averaged. Right panel: Cumulative distributions of individual SypHy puncta of all cells recorded. **D** The decay time constant of SypHy fluorescence decay was significantly increased in N-cadherin-CTF1 expressing neurons. Monoexponential fit of the average time course of SypHy signals from all puncta of a given cell (**A**) was used. Individual values for each cell (left) and mean ± SEM (right) are shown. One-way ANOVA with Tuckey’s post hoc test; **P < 0.01.

**Fig. 3:**
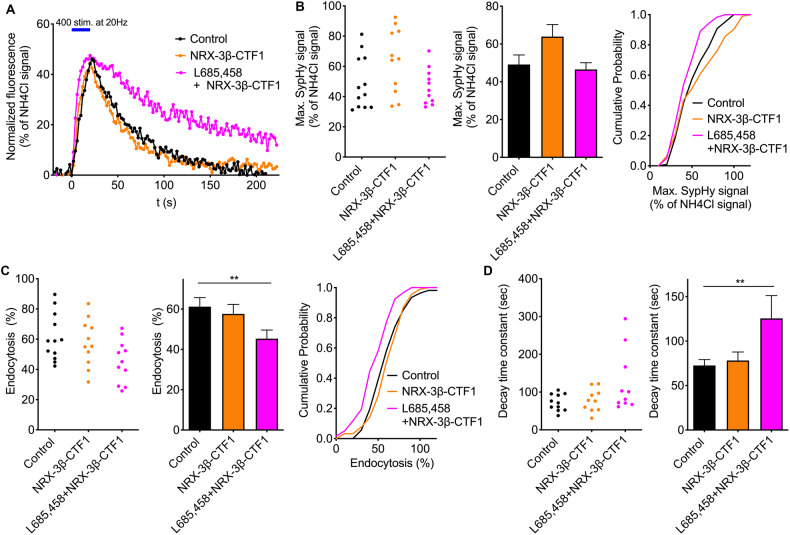
Overexpression of Neurexin3β-CTF1 did not affect synaptic vesicle endocytosis. **A** Time courses of SypHy fluorescence changes (elicited by 400 stimuli at 20 Hz; indicated by blue bar) in control neurons (black trace), in Neurexin3β-CTF1 expressing neurons (orange trace), and in Neurexin3β-CTF1 expressing neurons with addition of L-685,485 (5 µM for 2 days; magenta trace). Individual SypHy puncta on a given neuron were averaged per cell and normalized to the NH_4_Cl signal. **B** The maximal SypHy fluorescence signal did not significantly differ between control and Neurexin3β-CTF1 expressing neurons. Left and middle panel: SypHy puncta of each cell (control: *n* = 12; Neurexin3-CTF1 expressing: *n* = 11; Neurexin3β-CTF1 expressing with addition of L-685,485: *n* = 11) were averaged. Individual values for each cell (left) and mean ± SEM (middle) are shown. Right panel: cumulative distributions of individual SypHy puncta of all cells recorded (control: *n* = 108 puncta; Neurexin3β-CTF1 expressing: *n* = 92 puncta; Neurexin3β-CTF1 expressing with addition of L-685,485: *n* = 119 puncta). **C** The percent loss of SypHy signal (% endocytosis) 90 s after end of stimulation was not altered in Neurexin3β-CTF1 expressing neurons. Addition of L-685,485 served as positive control. Left and middle panel: SypHy puncta of each cell were averaged. Right panel: cumulative distributions of individual SypHy puncta of all cells recorded. **D** The decay time constant of SypHy fluorescence decay was not altered in Neurexin3β-CTF1 expressing neurons. Addition of L-685,485 served as positive control. Monoexponential fit of the average time course of SypHy signals from all puncta of a given cell (**A**) was used. Individual values for each cell (left) and mean ± SEM (right) are shown. One-way ANOVA with Tuckey’s post hoc test; **P < 0.01.

To address this question, we overexpressed the major CTFs of the specific synaptic adhesion proteins N-cadherin and Neurexin3β (N-cadherin-CTF1 and Neurexin3β-CTF1 [30]) in cortical neurons in microisland cultures. As previously reported [30, 31], Neurexin3β-CTF1 is the major Neurexin3β-CTF generated following the proteolytic processing of the full-length Neurexin3β by the sheddases ADAM10 and ADAM17. N-cadherin-CTF1 or Neurexin3β-CTF1 was co-expressed with SypHy and the transfection marker DsRed2 in individual neurons (transfection at 10 DIV). After 2–4 days, SypHy imaging was performed at autaptic sites after 12–14 DIV (Figs. 2A and 3A). To further study whether additional effects on synaptic vesicle endocytosis can be induced by concomitant γ-secretase blockade, we performed pharmacological inhibition of γ-secretase by L-685,485 on top of CTF overexpression.

The maximal SypHy fluorescence increase at the end of the electrical stimulation (400 stimuli at 20 Hz) did not significantly differ between control neurons (SypHy and DsRed2 expression only) and N-cadherin-CTF1 overexpressing neurons (Fig. 2B). We further analyzed the synaptic vesicle endocytosis-related SypHy fluorescence decay following the end of stimulation. Interestingly, at 90 s after the end of stimulation the relative SypHy signal loss was significantly reduced in N-cadherin-CTF1 expressing neurons (Fig. 2C). Accordingly, analysis of the SypHy fluorescence decay kinetics by monoexponential fitting revealed that the mean decay time constant was significantly increased in N-cadherin-CTF1 expressing neurons (Fig. 2D). These data further strengthen our previous results obtained in independent experiments [28] and indicate a slowing of synaptic vesicle endocytosis by N-cadherin-CTF1 overexpression. Moreover, the slowing of synaptic vesicle endocytosis observed following N-cadherin-CTF1 overexpression was not significantly increased by the additional inhibition of γ-secretase by L-685,485 (Fig. 2C, D). The latter result suggests that an increase in N-cadherin-CTF1 alone might be sufficient to explain the global slowing effects with inhibition of γ-secretase activity (described in Fig. 1).

In sharp contrast to the effects observed with overexpression of N-cadherin-CTF1, overexpression of Neurexin3β-CTF1 [30, 31] did not result in any significant changes in electrical stimulation-induced SypHy fluorescence signals (Fig. 3A–D). Neither the relative SypHy signal loss at 90 s after the end of stimulation (Fig. 3C), nor the mean decay time constant of the SypHy fluorescence decay (Fig. 3D) were significantly altered. However, addition of L-685,458 as a positive control resulted in a clear slowing of endocytosis (Fig. 3). These results indicate that overexpression of Neurexin3β-CTF1 does not induce a delay of synaptic vesicle endocytosis. In summary, our results with overexpression of specific synaptic adhesion protein CTFs suggest that N-cadherin-CTF1, but not Neurexin3β-CTF1, is of particular importance in the delay of synaptic vesicle endocytosis that is induced by γ-secretase inhibition.

### Slowing of synaptic vesicle endocytosis resulted in enhanced Aβ_42_ synaptotoxicity

We previously reported that increased expression of N-cadherin-CTF1 by γ-secretase inhibition resulted in an enhancement of Aβ induced synaptotoxicity [1]. Here, we further addressed, whether this enhanced Aβ synaptotoxicity can be specifically attributed to the slowing of synaptic vesicle endocytosis that is induced by N-cadherin-CTF1. Therefore, we partially inhibited synaptic vesicle endocytosis indirectly by pharmacological inhibition of γ-secretase (L-685,485, see Fig. 1) or directly by the addition of a low concentration of the endocytosis inhibitor dynasore (20 µM, see Supplementary Fig. 2 for partial inhibition) and analyzed whether a simultaneous short-term application of synthetic Aβ_42_ (1 µM for 2 days, without toxic effects in controls) under conditions of slowing of synaptic vesicle endocytosis results in enhanced synaptotoxicity.

**Fig. 4:**
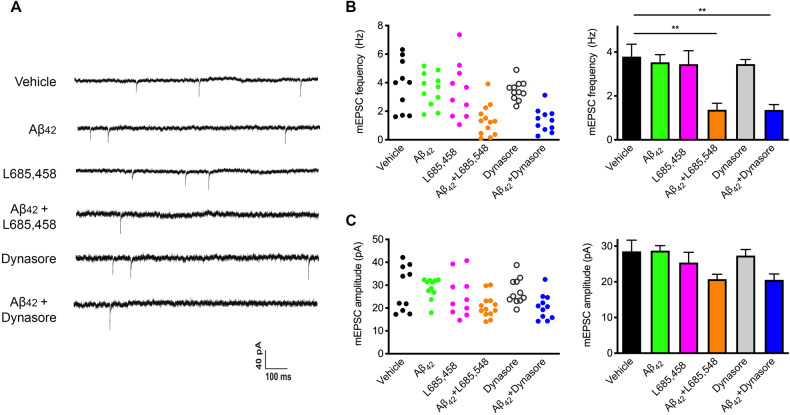
Slowing of synaptic vesicle endocytosis by partial inhibition resulted in enhanced Aβ_42_ synaptotoxicity. **A**–**C** Synaptotoxicity induced by short-term application of synthetic Aβ_42_ (1 µM) for 2 days to cultured cortical neurons was monitored by patch-clamp recordings of AMPA mEPSCs. L-685,485 (5 µM) or dynasore (20 µM) were co-applied to partially inhibit synaptic vesicle endocytosis (resulting in slower kinetics). **A** Example traces of AMPA mEPSCs recorded at −60 mV holding potential in the presence of 1 µM TTX and 10 µM gabazine, and under the experimental conditions indicated in front of each trace. **B** Quantitative analysis of AMPA mEPSC frequencies. Left: data for individual cells; *n* = 10/11/10/13/11/11 cells. Right: means ± SEM. **C** Quantitative analysis of AMPA mEPSC mean amplitudes. Note that the combination of two non-toxic conditions resulted in an enhanced synaptotoxicity. One-way ANOVA with Tuckey’s post hoc test. **P < 0.01.

Aβ-induced synaptotoxicity was studied by patch-clamp recordings of miniature excitatory postsynaptic currents (mEPSCs) in cultured cortical neurons (mass cultures at 12–14 DIV). As a control experiment, we first confirmed that a long-term 4 days incubation of cultured cortical neurons with our Aβ_42_ preparation was synaptotoxic. As expected, this standard application scheme resulted in a strong reduction in mEPSC frequency and mean amplitude (Supplementary Fig. 3). Next, we performed a short-term application of the same Aβ_42_ preparation for only 2 days that alone did not result in significant changes in mEPSC frequeny and mean amplitude (Fig. 4). We then combined this non-synaptotoxic short-term Aβ_42_ application with treatments inducing a slowing of synaptic vesicle endocytosis.

As expected from our previous report using 7PA2 supernatant as an Aβ preparation [1], indirectly inducing a delay of synaptic vesicle endocytosis with non-synaptotoxic L-685,485 (5 µM for 2 days) resulted in a clear synaptotoxicity effect of a simultaneous 2 days synthetic Aβ_42_ application, as indicated by a significantly reduced mEPSc frequency (Fig. 4). Moreover, mEPSC mean amplitudes were not significantly altered, but showed a trend towards reduced amplitudes (Fig. 4).

Next, we directly induced a slowing of synaptic vesicle endocytosis by addition of a low concentration of the endocytosis inhibitor dynasore (20 µM for 2 days). Most interestingly, this resulted also in a synaptotoxicity effect of a simultaneous 2 days Aβ_42_ application. Again, the combination of two non-toxic conditions (dynasore alone and 2 days Aβ_42_ alone) resulted in clear synaptotoxicity, as indicated by a significantly reduced mEPSC frequency and a trend to reduced amplitudes (Fig. 4).

**Fig. 5:**
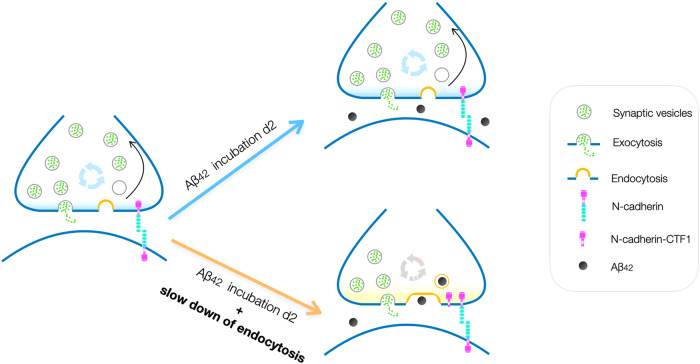
Model for enhanced synaptotoxicity caused by the combination of Aβ oligomers and slowed synaptic vesicle endocytosis. **Left:** Transsynaptic N-cadherin interactions have a crucial role in enabling fast synaptic vesicle endocytosis. Upper right: Fast synaptic vesicle endocytosis does not allow for efficient uptake of toxic Aβ oligomers during a 2 days incubation, because Aβ oligomer binding to its intraluminal membrane receptors might be a very slow process. Lower right: Disturbing transsynaptic N-cadherin interactions by increased presence of N-cadherin-CTF1 leads to a slow down of synaptic vesicle endocytosis. Slow down of endocytosis in turn increases Aβ oligomer uptake, because slow Aβ binding can now take place before vesicle endocytosis is completed. Intracellular Aβ might then induce synaptotoxic effects.

Altogether, our results demonstrate an enhanced synaptotoxicity of Aβ_42_ under conditions of partial inhibition of synaptic vesicle endocytosis. This might mechanistically underlie the previously described enhancement of Aβ_42_ synaptotoxicity that is induced by N-cadherin-CTF1 overexpression [1]. As depicted in Fig. 5, an increase in N-cadherin-CTF1 induces a delay in synaptic vesicle endocytosis and the latter in turn underlies an enhanced Aβ_42_ synaptotoxicity.

## Discussion

In this study, we provide new insights into the subcellular and molecular mechanisms that underlie the enhancement of Aβ synaptotoxicity by a proteolytic fragment of the synaptic adhesion protein N-cadherin (N-cadherin CTF1) [1]. We found that general inhibition of γ-secretase activity led to an impairment of synaptic vesicle endocytosis resulting in a slowing of endocytosis kinetics. Because γ-secretase has a very large number of substrates (CTFs of single transmembrane proteins) [32], this effect might be attributed to an increased expression level of several different CTFs. Here, we further strengthened our previous result [28] that increased expression of N-cadherin CTF1 led to a slowing of endocytosis kinetics. In the current study, this effect on endocytosis was observed selectively for N-cadherin CTF1, but not for Neurexin3β-CTF1, another important synaptic adhesion protein CTF [30, 31]. Moreover, this effect was comparable to the slowing of endocytosis induced by general inhibition of γ-secretase suggesting that N-cadherin CTF1 plays a major role.

In a second round of experiments, we addressed whether a slowing of vesicle endocytosis in general leads to an enhancement of Aβ synaptotoxicity. Most interestingly, we found that partial inhibition of endocytosis by a low concentration of the specific endocytosis blocker dynasore resulted in enhanced Aβ synaptotoxicity as indicated by a reduced frequency of miniature EPSCs upon short-term Aβ application.

Our findings led us to propose the model depicted in Fig. 5. In this model, we propose that a critical amount of intracellular Aβ oligomer accumulation is required to induce synaptotoxic damage to organelles in subcellular compartments such as presynaptic mitochondria. Because binding of Aβ oligomers to the luminal side of vesicle membranes (only accessible upon exocytosis) might be a very slow process, normal vesicle endocytosis kinetics might be too fast to enable sufficient Aβ uptake (Fig. 5, upper right). Expression of N-cadherin CTF1 leads to a slowing of endocytosis resulting in a longer exposure of luminal vesicle membranes to the extracellular space. This increases the time interval for binding of Aβ oligomers before endocytosis takes place and thus might enable increased Aβ uptake (Fig. 5, lower right). In accordance with this model, N-cadherin has been well described to positively modulate synaptic vesicle endocytosis [28, 33, 34], and N-cadherin CTF1 is well known to inhibit N-cadherin function most likely by competing with full-length N-cadherin binding to catenins [35].

The role of intracellular Aβ oligomers in AD still remains largely unclear. Impairment of mitochondrial functions has been described to be a major subcellular target of toxic Aβ species inside neurons [24, 25, 36–38]. This might be of particular importance in Aβ-induced presynapse degeneration, because mitochondria are accumulated at presynaptic sites [22, 39]. Presynaptic mitochondria are well known for their essential role in synapse function and synapse stability [40]. The deleterious effects of intracellular Aβ on presynapses might start with an impairment of normal mitophagy [19, 21, 41, 42] leading to an accumulation of defective mitochondria [24]. Because mitophagy and autophagy are closely related subcellular processes, the emerging effects of Aβ on autophagy [20, 41, 43] might be also of relevance in presynapse degeneration.

The occurrence of intracellular Aβ appears to require reuptake of extracellular Aβ oligomers by endocytosis of surface membrane [26, 44–46]. The endocytosis of synaptic vesicles might therefore be a major mechanism of Aβ reuptake at presynaptic sites. Because the synaptic adhesion protein N-cadherin has been demonstrated to modulate synaptic vesicle endocytosis [28, 33, 34], the slow down of vesicle endocytosis by N-cadherin CTF1 is in line with the physiological function of N-cadherin. Moreover, known AD genetic risk factors such as BIN1 and PICALM are mechanistically involved in the regulation of endocytosis processes [47, 48]. This suggests that dysregulation of endocytosis plays an important role in AD pathogenesis.

As described in our previous paper [1], N-cadherin CTF1 expression appears to be increased at least in a subset of AD patients. The slowing of synaptic vesicle endocytosis by N-cadherin CTF1 might therefore be of relevance for the pathomechanisms of AD in affected individuals. This is further supported by the fact that several human genetic risk factors are proteins related to endocytosis [47, 48]. The accumulation of N-cadherin CTF1 in AD patients might be caused by a dysfunction of γ-secretase leading to a less efficient further breakdown of N-cadherin CTF1 [1, 49–53]. Alternatively, the increase in N-cadherin CTF1 might be explained by an increased activity of α-secretase (ADAM10) leading to an increased proteolysis of full-length N-cadherin and thus an increased N-cadherin CTF1 production [54–57]. Altogether, our findings suggest that the delay in synaptic vesicle endocytosis induced by increased N-cadherin CTF1 levels plays an important role in synaptotoxicity caused by intracellular Aβ oligomers in AD.

## Materials and methods

### Cell culture

Because we wanted to express CTFs of synaptic adhesion proteins both pre- and postsynaptically, we used microisland cultures of mouse cortical neurons in this study. In microisland cultures [58], one or a few dissociated neurons are cultured on top of a co-cultured astrocyte serving as a “microisland” substrate. In such microisland cultures, neurons form synaptic contacts on their own dendrites (autapses) [58], because axon growth is confined to the vicinity of the cell body (see Supplementary Fig. 1A). Autaptic microisland co-cultures of dissociated astrocytes and neurons from cortices of C57/BL6 wildtype mice were done as described previously [28, 33]. Briefly, P0 cortical tissue was mechanically dissociated after trypsin treatment to obtain cortical astrocytes, which were then long-term cultured to form a confluent monolayer in BME medium (Gibco) containing 10% FBS, L-glutamine (2 mM), glucose (20 mM), insulin transferrin selenium A (ITS, 1%), and penicilline-streptomycine (1%). Then, these astrocytes were re-dissociated and cultured on glass coverslips for additional 5–7 days in BME medium to obtain glial microislands. Neurons were prepared by dissociating (after trypsin for 5 min) cortical tissue from cortices of E18-19 mouse fetuses. Cortical neurons were seeded at a density of 20,000–30,000 cells per culture dish on glial microisland cultures, and were co-cultured in Neurobasal (NB) medium (Gibco) including 2% B27 supplement, 0.5% Glutamax-I supplement (Gibco) and 1% penicilline-streptomycine. Further incubation and maintenance of these autaptic microisland co-cultures was done for 12–16 days at 37 °C in a humidified incubator with 5% CO_2_.

Primary neuronal cultures were performed as described previously [28, 33]. Briefly, cortical tissue from E18-19 fetuses of C57/BL6 wildtype mice was subjected to a 5 min trypsin treatment and then was mechanically dissociated. Dissociated neurons were plated at a density of 20,000–30,000 cells per culture dish on poly-L-ornithine pre-coated glass coverslips in NB medium wth supplements (see above). These cortical neuron cultures were maintained for an additional 12–14 days at 37 °C in a humidified incubator with 5% CO_2_.

### Transfection and plasmids

Transfection of individual neurons in autaptic microisland co-cultures was performed by using magnetic nanoparticles (NeuroMag; OZ Biosciences) as described previously [28, 33]. In brief, DNA/NeuroMag complexes with magnetic nanoparticles and plasmid DNA were prepared in NB medium without any supplements and incubated for 20 min at room temperature. Plasmids used were SypHy-A4 (from Dr. L. Lagnado, Cambridge, UK, via addgene), pDsRed2-N1 (Clontech), pcDNA3.1-Neurexin3β-CTF1 (from Dr. P.C. Fraering) and pcDNA3.1-FLAG-N-cadherin-CTF1 [1]. More precisely, Neurexin3β-CTF corresponds to the a.a. residues 349–432 of the Human NRXN3β sequence (Q9HDB5-2, as listed in the Uniprot database [30, 31]). Next, DNA/NeuroMag complexes were added to the microisland co-cultures in a 6-well plate for 30 min at 37 °C and subjected to an oscillating magnetic field (Magnetofection TM, magnefect LT; nano Therics) to enhance the transfection efficiency.

### Synaptophysin-pHluorin (SypHy) imaging and data analysis

Synaptophysin-pHluorin (SypHy) imaging was used to monitor exocytosis and endocytosis of synaptic vesicles as described previously [28, 33]. Briefly, cortical neurons in autaptic microisland co-cultures were co-transfected with SypHy-A4 and DsRed2 (and in some experiments additionally with Neurexin3β-CTF1 or N-cadherin-CTF1), and the SypHy imaging experiments were carried out 2 to 3 days after transfection. After transfering cells to a stimulation chamber (Live Cell Instruments) on the stage of an Axiovert 200 M microscope (Zeiss), electrical stimulation was used to induce vesicle exocytosis in standard extracellular solution (in mM: 136 NaCl, 2.5 KCl, 2 CaCl_2_, 1.3 MgCl_2_, 10 HEPES, 10 Glucose, pH = 7.3). Recurrent network activity was prevented by adding DNQX (10 μM) and DL-AP5 (50 μM) to the extracellular medium.

To determine the maximal SypHy fluorescence signal, the total vesicle pool at all autapses/synapses of a transfected neuron was alkalized by adding an extracellular solution with a high concentration of NH_4_Cl (in mM: 50 NH_4_Cl, 86 NaCl, 2.5 KCl, 2 CaCl_2_, 1.3 MgCl_2_, 10 HEPES, 10 Glucose, pH = 7.3) at the end of each experiment (Supplementary Fig. 1). The DsRed2 fluorescence images (transfection marker) were superimposed on the maximal SypHy fluorescence signal to identify autaptic sites. The maximal SypHy fluorescence signal was used for normalization of SypHy fluorescence signal time courses. Fluorescence images were taken at time intervals of 2 s to record the SypHy fluorescence signal changes caused by synaptic vesicle cycling induced by electrical stimulation. MetaVue software (Visitron Systems) was used to determine the fluorescence intensities at individual SypHy puncta. Regions of interest (ROIs) were defined around individual SypHy puncta at autaptic sites. The average SypHy fluorescence intensity within a ROI was quantitatively determined at each time point to obtain SypHy fluorescence transients. All individual SypHy fluorescene transients of a given cell were normalized to the maximal SypHy signal, and were averaged for each cell for quantitative comparison.

### Western blots

According to previously established standard methods [1, 59], samples of cultured hippocampal neurons were homogenized and processed for protein analysis. The protein concentrations were quantified using the BCA-kit (Bio-Rad). Each sample (15 μg from cell lysates) was run on a 4–12% Bis-Tris gel (Invitrogen) and protein bands were transferred (duration 1.5 h) to a 0.2 mm Nitrocellulose membrane at 400 mA for subsequent Western blot analysis. The following antibodies were used: mouse anti-N-cadherin (1:400; BD Transduction Laboratories), rabbit anti-Neuroligin1 (1:1000; Synaptic Systems), rabbit anti-Neurexin 1/2/3 (1:1000; Synaptic Systems), mouse anti-beta actin (1:10000; Sigma), goat anti-mouse IgG HRP-conjugated (Sigma) or goat anti-rabbit IgG HRP-conjugated (Sigma).

### Synthetic Aβ_42_ oligomer preparation

After being dissolved in 1,1,1,3,3,3-Hexafluoro-2-propanol (HFIP), synthetic amyloid-β_1-42_ (Aβ_42_, from Bachem) was vortexed and sonicated in an ultrasonic water bath. Following that, HFIP dissolved Aβ_42_ and HFIP vehicle aliquots were kept at −80 °C. These aliquots were freshly prepared for experiments by evaporating them under a soft stream of nitrogen. After complete evaporation they were redissolved (at a final concentration of 5 μM) with NB + B27 medium at 4 °C for 48 h. Finally, cultured cortical neurons were treated with 1 μM Aβ_42_ or HFIP vehicle for two days prior to experimental analysis.

### Electrophysiology and data analysis

Mass cultures of cortical neurons incubated in the presence (for 2 days prior to recording) or absence of synthetic Aβ_42_ (see above, 1 µM), the γ-secretase inhibitor L-685,458 (Tocris, 5 μM) or dynasore (Tocris, 20 μM) were subjected to whole-cell patch-clamp recordings using an Axopatch 200B patch-clamp amplifier and pClamp 11 software (Molecular Devices, SanJose, CA) as described previously [59, 60]. Patch pipettes were filled with an intracellular solution containing (in mM): 110 KCl, 20 HEPES, 10 EGTA, 0.25 CaCl_2_ with pH adjusted to 7.3. The standard extracellular solution contained (in mM): 130 NaCl, 3 KCl, 2.5 CaCl_2_, 1 MgCl_2_, and 20 HEPES, with pH adjusted to 7.3. AMPA receptor-mediated miniature excitatory postsynaptic currents (AMPA mEPSCs) were recorded at a holding potential of −60 mV in the presence of TTX (1 µM) and gabazine (10 µM). Quantitative analysis of AMPA mEPSCs was carried out using Mini Analysis software (Synaptosoft, Decatur, GA, USA).

### Statistics

All data are given as means ± SEM. The sample sizes (*n*) are given as numbers in the figure legends. *n* ≥ 10 (cells) was used for all experimemtal groups, and *n* represents biological replicates. All experiments were replicated in at least three cultures. Student’s *t* test and one-way ANOVA in combination with Tuckey’s post hoc test were used to determine statistical significance by using SigmaPlot 11 software (see also figure legends). *P* values are given in the figure legends.

### Supplementary information

Supplementary Figure 1

Supplementary Figure 2

Supplementary Figure 3

Uncropped Westerns

## Data Availability

Original data will be made available to other researchers on request.
