# Supplementary material for: A delay in vesicle endocytosis by a C-terminal fragment of N-cadherin enhances Aβ synaptotoxicity
Source: Cell Death Discov. 2023 Dec 8;9:444. doi: 10.1038/s41420-023-01739-w (PMC10703901; doi:10.1038/s41420-023-01739-w)
Supplement: Supplementary file 1 — Supplementary Figure 1 [file 41420_2023_1739_MOESM1_ESM.pdf]

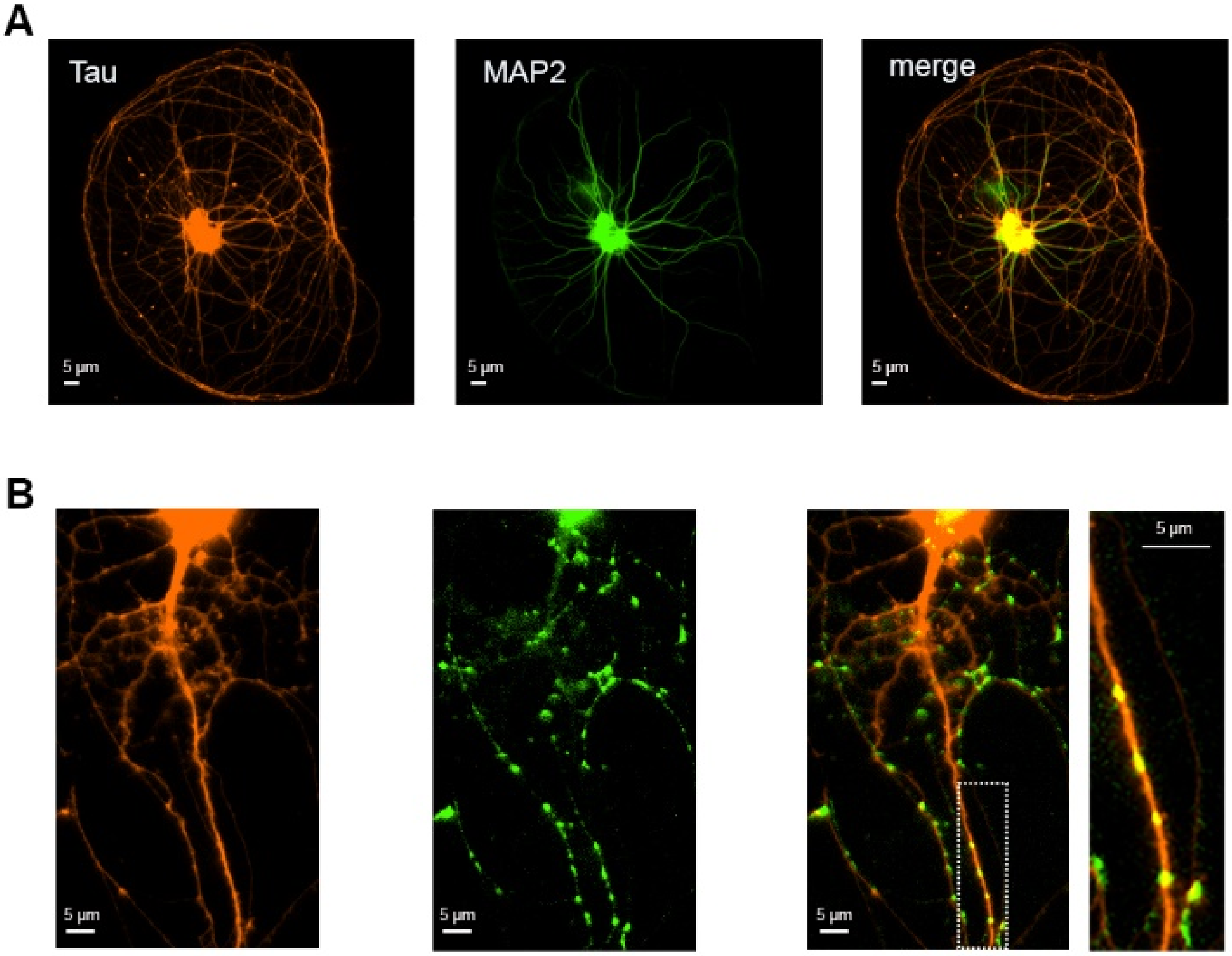

Supplementary Figure 1: Microisland cell cultures. (**A**) Mouse cortical neurons in microisland culture. 12-14 days in vitro. Left: Tau staining of axons. Middle: MAP2 staining of dendrites. Right: Overlay (merge) indicating autaptic contacts. The following primary antibodies were used: Anti-MAP2 (Chicken polyclonal, 1:1000, Abcam); Anti-Tau (Monoclonal mouse, 1:1000, Synaptic Systems). The secondary antibodies included AF555 goat anti-mouse (1:1000, Life Technologies) and AF488 goat anti-chicken (1:1000, Life Technologies). (**B**) Identification of autaptic contacts for SypHy imaging. Individual neurons in microisland culture were co-transfected with DsRed2 (transfection marker) and SypHy. Left: DsRed2 fluorescence visualising a transfected neuron. Middle: $NH4^+$ induced SypHy punctate fluorescence (at end of experiments). Right: Overlay of DsRed2 and SypHy fluorescence enabling identfication of autaptic contacts on large caliber dendrites. White box is shown enlarged.
