# Supplementary material for: A delay in vesicle endocytosis by a C-terminal fragment of N-cadherin enhances Aβ synaptotoxicity
Source: Cell Death Discov. 2023 Dec 8;9:444. doi: 10.1038/s41420-023-01739-w (PMC10703901; doi:10.1038/s41420-023-01739-w)
Supplement: Supplementary file 2 — Supplementary Figure 2 [file 41420_2023_1739_MOESM2_ESM.pdf]

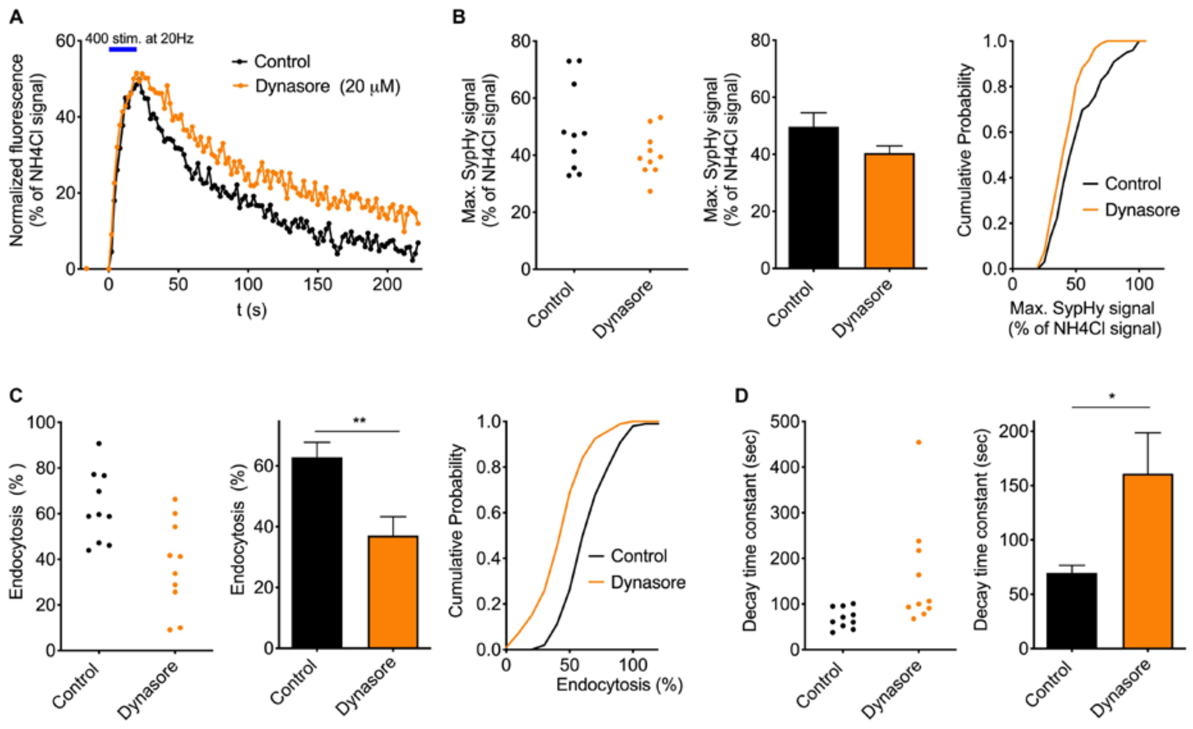

Supplementary Figure 2: Slowing of synaptic vesicle endocytosis upon partial inhibition by dynasore (20 µM). (**A**) Time courses of SypHy fluorescence changes (elicited by 400 stimuli at 20 Hz; indicated by blue bar) in control neurons (black trace) and in the presence of dynasore (20 µM; orange trace). Individual SypHy puncta on a given neuron were averaged per cell and normalized to the NH_4Cl signal. (**B**) The maximal SypHy fluorescence signal did not significantly differ between controls and dynasore application. Left and middle panel: SypHy puncta of each cell (control: n=10; addition of dynasore: n=10) were averaged. Individual values for each cell (left) and mean ± SEM (middle) are shown. Right panel: Cumulative distributions of individual SypHy puncta of all cells recorded (control: n=99 puncta; addition of dynasore: n=93 puncta). (**C**) The percent loss of SypHy signal (% endocytosis) 90 seconds after end of stimulation was significantly reduced in the presence of dynasore. Left and middle panel: SypHy puncta of each cell were averaged. Right panel: Cumulative distributions of individual SypHy puncta of all cells recorded. (**D**) The decay time constant of SypHy fluorescence decay was significantly increased in the presence of dynasore. Monoexponential fit of the average time course of SypHy signals from all puncta of a given cell (A) was used. Individual values for each cell (left) and mean ± SEM (right) are shown.

Student´s t-test; * P<0.05; ** P < 0.01.
