# Supplementary material for: A delay in vesicle endocytosis by a C-terminal fragment of N-cadherin enhances Aβ synaptotoxicity
Source: Cell Death Discov. 2023 Dec 8;9:444. doi: 10.1038/s41420-023-01739-w (PMC10703901; doi:10.1038/s41420-023-01739-w)
Supplement: Supplementary file 3 — Supplementary Figure 3 [file 41420_2023_1739_MOESM3_ESM.pdf]

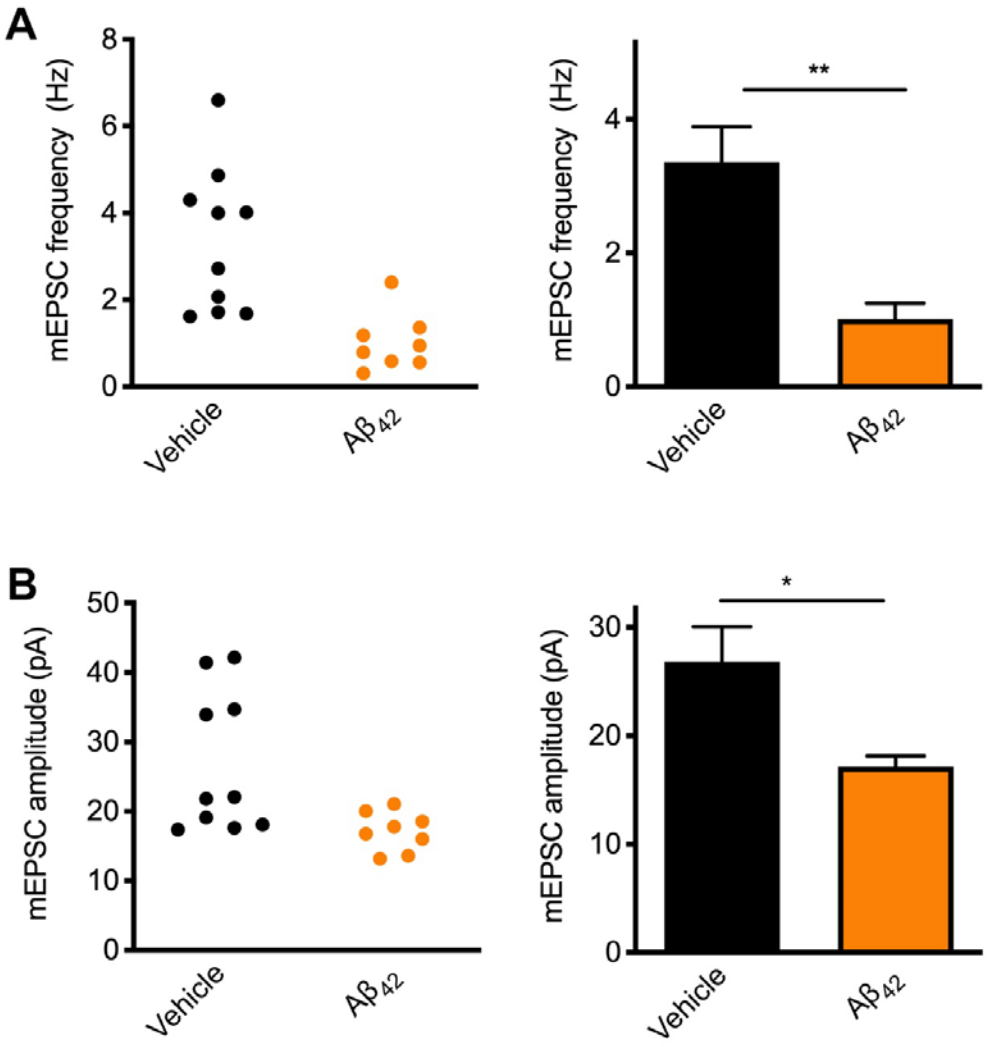

Supplementary Figure 3: Synaptotoxic effects of 4 days application of synthetic Aβ_{42} in cultured cortical neurons. Synaptotoxic effects were monitored by patch-clamp recordings of AMPA mEPSCs (at 12-14 DIV; holding potential: -60 mV; 1 µM TTX and 10 µM gabazine added). (**A**) Quantification of AMPA mEPSC frequencies. n = 10/8 cells. Left: data from individual cells. Right: means ± SEM. (**B**) Quantification of AMPA mEPSC mean amplitudes. Left: data from individual cells. Right: means ± SEM. Student´s t-test; * $P < 0.05$; ** $P < 0.01$.
