# Supplementary figures and images for: A delay in vesicle endocytosis by a C-terminal fragment of N-cadherin enhances Aβ synaptotoxicity
Source: Cell Death Discov. 2023 Dec 8;9:444. doi: 10.1038/s41420-023-01739-w (PMC10703901; doi:10.1038/s41420-023-01739-w)

## Supplementary information

### Western blots (uncropped)

used for Figure 1:

Figure 1E

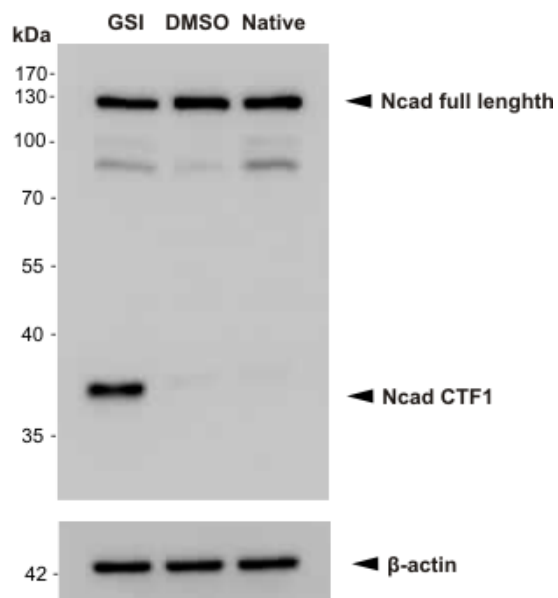

Figure 1F

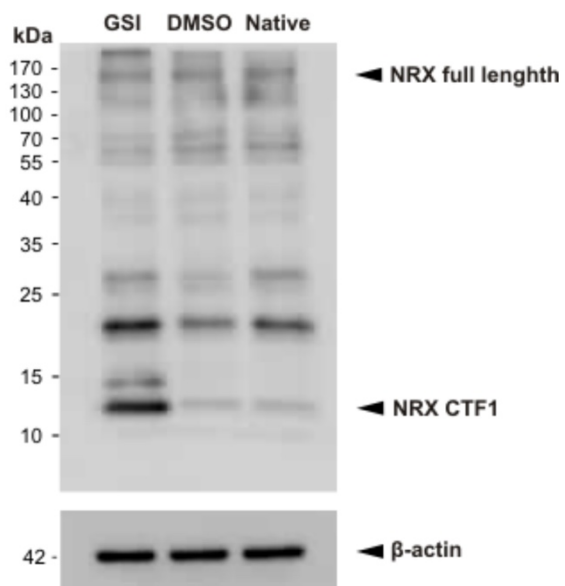

Figure 1G

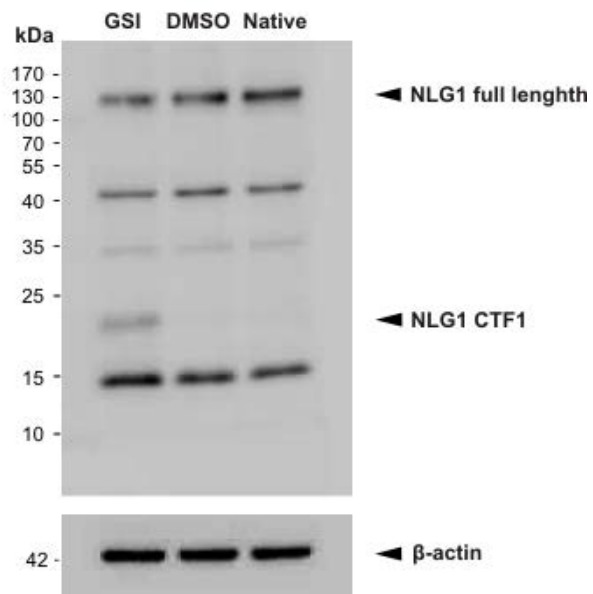

Supplement: Supplementary file 4 — Uncropped Westerns [file 41420_2023_1739_MOESM4_ESM.pdf]
